# Bacteriophage therapy for inhibition of multi drug‐resistant uropathogenic bacteria: a narrative review

**DOI:** 10.1186/s12941-021-00433-y

**Published:** 2021-04-26

**Authors:** Zahra Chegini, Amin Khoshbayan, Soheil Vesal, Alireza Moradabadi, Ali Hashemi, Aref Shariati

**Affiliations:** 1 Department of Microbiology, School of Medicine, Iran University of Medical Sciences, Tehran, Iran; 2 Department of Molecular Genetics, Faculty of Basic Sciences and Advanced Technologies in Biology, University of Science and Culture, Tehran, Iran; 3 Department of Medical Laboratory Sciences, Khomein University of Medical Sciences, Khomein, Iran; 4 Department of Microbiology, School of Medicine, Shahid Beheshti University of Medical Sciences, Tehran, Iran

**Keywords:** Phage therapy, Urine, Multi‐drug resistant, Urinary tract infection

## Abstract

Multi-Drug Resistant (MDR) uropathogenic bacteria have increased in number in recent years and the development of new treatment options for the corresponding infections has become a major challenge in the field of medicine. In this respect, recent studies have proposed bacteriophage (phage) therapy as a potential alternative against MDR Urinary Tract Infections (UTI) because the resistance mechanism of phages differs from that of antibiotics and few side effects have been reported for them. *Escherichia coli*, *Klebsiella pneumoniae*, and *Proteus mirabilis* are the most common uropathogenic bacteria against which phage therapy has been used. Phages, in addition to lysing bacterial pathogens, can prevent the formation of biofilms. Besides, by inducing or producing polysaccharide depolymerase, phages can easily penetrate into deeper layers of the biofilm and degrade it. Notably, phage therapy has shown good results in inhibiting multiple-species biofilm and this may be an efficient weapon against catheter-associated UTI. However, the narrow range of hosts limits the use of phage therapy. Therefore, the use of phage cocktail and combination therapy can form a highly attractive strategy. However, despite the positive use of these treatments, various studies have reported phage-resistant strains, indicating that phage–host interactions are more complicated and need further research. Furthermore, these investigations are limited and further clinical trials are required to make this treatment widely available for human use. This review highlights phage therapy in the context of treating UTIs and the specific considerations for this application.

## Introduction

Urinary Tract Infections (UTIs) constitute one of the most important concerns among medical experts and patients that are linked to almost 40% of the cases of nosocomial infections in acute care hospitals [1, 2]. Notably, these infections are reported as one of the most common bacterial infections that affecting about 150 million people every year worldwide. UTI is one of the most common reason of morbidity and mortality in the elderly, accounting for 15.5 and 6.2% of hospitalization and deaths of people aged 65 years or older, respectively [3–5]. Women are more susceptible to UTIs than men due to behavioral factors, their anatomy, and practice such as use of diaphragm and spermicides [6]. UTIs may affect women at any age, especially women with frequent sexual activities and women of childbearing age as well as old men and infant boys [2]. UTIs are classified as either lower (confined to the bladder) or upper (pyelonephritis) and they can be clinically divided into two forms: uncomplicated and complicated. Uncomplicated form represents the conditions when patients exhibit a healthy status previous to the infection without non-catheterized, non-pregnant, and no structural abnormalities. On the other hand, UTI is reported as complicated when patients are immunocompromised or experience risk factors such as pregnancy and urinary retention [1, 7]. *Escherichia coli* is the most common (80%) cause of the uncomplicated UTI and catheter-associated infections [8]. Other common bacteria in UTIs include *Proteus* species, *Klebsiella pneumoniae, Staphylococcus aureus, Staphylococcus saprophyticus*, and *Enterococci* and *Enterobacter* [2, 9]. *Pseudomonas aeruginosa* and *Acinetobacter baumannii* are rarely isolated from UTIs, but the epidemic potential of Multi-Drug Resistant (MDR) pathogens and high ability to cause septicemia makes these bacteria quite important [10]. Prophylaxis of UTIs or extensive and unlimited use of routine antibiotics leads to the emergence of MDR bacteria. Therefore, treating UTIs due to antibiotic resistance is becoming more difficult every day [5].

One of the main features of uropathogenic bacteria that makes them more resistant to the host immune system and chemical antibiotics is their capacity to form single- or mixed-species biofilm. This structure settles on both abiotic and biological surfaces such as indwelling urinary catheters and is characterized by low susceptibility to antibiotics and hard to remove even if the catheter is detached [1, 11]. Thus, the high prevalence of MDR bacteria has severely restricted the use of antibiotics in recent years. Furthermore, a long course of antibiotic therapy often causes toxicity in the patients, arising antibiotic resistance and affected natural microbiota. So, interest in other treatments like phage therapy is increasing. In this context, given the phages are self-replicating, they cannot infect eukaryotic cells and their mechanisms of resistance are different from those of antibiotics [12, 13]. Various studies suggests phage therapy has the potential to be used as either an alternative or a supplement to antibiotic treatments [14, 15]. Bacteriophage depolymerase plays an important role in the degradation of biofilm Extracellular Polymeric Substances (EPS) substrate, promoting phage penetration into the biofilm and leading to bacterial cell lysis. Indeed, this enzyme is expressed on the surface of phage capsids or produced by host cells during phage replication and it helps phages to adsorb, attack, and decompose bacterial host [16]. Furthermore, phages at the end of the lytic cycle produce endolysins. Endolysins are phage enzymes that cleave peptidoglycans, i.e., the main component of the bacterial cell wall, and they are antibacterial agents owing to their special mode of action and highly particular activities against bacteria [17]. Noteworthy, phage therapy can be divided into two categories: monophage and polyphage therapy. Two or more phages mix and they cover various bacterial hosts in a single product as a phage cocktail which is typically more effective in interfering in bacterial infections [18, 19].

Furthermore, recent studies have reported that despite the presence of the different factors such as biofilms and other antibiotic resistance factors in UTIs, phages along with other antimicrobial strategies may be effective in preventing and treating these infections by increasing the synergistic effect [20, 21]. In this respect, the present review paper is an attempt at investigating more comprehensively the effectiveness of phage therapy in treating UTIs caused by MDR-uropathogenic bacteria.

## *Escherichia coli*

*Escherichia coli* is one of the most common human pathogens and induces infection in different organs of the body including enteritis, septicemia, neonatal meningitis, and UTI [22, 23]. The most frequent extra-intestinal *E. coli* infection is UTI and by far, the most common pathogen that induces UTI is Uropathogenic *E. coli* (UPEC) [24]. Moreover, the growing prevalence of MDR UPEC strains has complicated UTIs treatment and led to rise of costs and extended hospital stays [25]. Nowadays, phage therapy is considered a potential choice for treating the resistance of UPEC [13]. In this context, VB_ecoS-Golestan is reported as an alternative treatment for inhibiting UPEC. Among investigated isolates, 78.8% were MDR, of which 56% exhibited sensitivity to the lytic activity of this phage. This phage is characterized by broad host range specificity in MDR isolates; besides, none of the lysogenic mediated genes was found in this phage genome. Therefore, the authors suggested that phage therapy could be a promising approach to the treatment of MDR *E. coli*. However, high specificity towards *E. coli* may be a restricting factor in applying the phage to treatment of multiple-species UTI [26].

Involvement of biofilms in most chronic infections is justified by their resistance to both antibiotics and host defenses. The need for effective treatments with an ability to penetrate the biofilm structure and destroy it can bring phages into attention as an antimicrobial agent to control and prevent biofilm formation [11]. Gu et al. characterized vB_EcoP-EG1(T7-like *Podoviridae* family) by large burst size, no toxic protein, very short lytic cycle, and broader host range on UPEC strains. This phage brings about promising outcome as it can infect 10 out 21 MDR UPEC. In addition, this phage successfully lyses the MDR UPEC and reduces the biofilm biomass in sensitive and MDR isolates. Furthermore, vB_EcoP-EG1 shows quite a strong lytic ability in both planktonic and biofilm forms of UPEC strains. In medical settings, the biofilm induces destructive damage during acute and chronic infections such as Catheter-Associated UTI (CAUTI) by UPEC, and the phage may have a potential to treat patients with biofilm-related UTI [27]. In another investigation, the authors reported three phages with activity against UPEC biofilm. All phages caused a reduction in biofilm biomass compared to the untreated group at both low and higher concentrations after 8 h of incubation. These results demonstrate that phages act in a nondose-dependent manner, working to their advantage when applied to in vivo condition in order to remove the biofilm. On the other hand, there was evidence that biofilm start to re-stablish itself after 24 h. This phenomenon could be linked to resistance development against the phages in bacteria [28].

Therefore, phages have a limited host range and the activation of resistance to phage in the biofilm community is one of the significant obstacles to successful phage therapy. These challenges can be overcome using phage cocktails and combination therapy of phage and antibiotics. It has already been shown that combination therapy not only reduces the number of bacteria but also relates to the management of phage-resistant bacteria levels [29]. Thus, some researches have investigated the effectiveness of combination therapy of phage and antibiotics in UPEC inhibition. In this respect, Moradpour et al. probed the synergic effect of phage and ampicillin against drug-resistant *E. coli* O157-associated UTI. The *E. coli* strain used in this study was resistant to ampicillin and had intermediate susceptibility to amoxicillin-clavulanic acid. Application of the phage resulted in keeping zones remarkably clear of ampicillin and amoxicillin-clavulanic acid in disk diffusion, reduced the overall growth of bacteria, and indicated the sensitivity of resistance phenotype in the inhibition zones. Further, the modified broth microdilution assay confirmed the phage-antibiotic synergy [30]. This synergistic effect may arise from stimulation of lytic phage growth in the presence of beta-lactams antibiotics. In fact, bacterial cells divide unsuccessfully and create very long filaments in the presence of beta-lactams, and this cell filamentation contributes to much faster phage assembly by simply lysing the cell. Filamentation induces perturbations in the peptidoglycan layer which may cause greater sensitivity to the activities of phage lysis [30, 31]. In addition, phage attack could alter the activity of efflux pump, causing increased sensitivity to multiple classes of antibiotic agents [32]. So, findings demonstrate the significant enhancement of bacteria killing in combination therapy, compared to each treatment alone. Similarly, recent research works have been directed at effects of combined use of phages and antibiotics on *E. coli* strain ATCC 13,706, being resistant to different antibiotics. Phage ECA2 and antibiotics was combined and subsequently, employed to evaluate the inactivation of *E. coli.* In this regard, the combination of phage and ciprofloxacin at the sub lethal concentration remarkably reduced the bacterial level compared to using each one alone. Besides, the presence of antibiotic at a sub-lethal concentration can control the formation of phage-resistant strain. However, at sub lethal doses, antibiotics create a phage-antibiotic synergistic effect and decrease the bacterial number, but only when bacteria are sensitive to the used antibiotics. Of note, ciprofloxacin hinders the enzyme topoisomerase II (DNA gyrase) of the bacteria and, subsequently, interferes in nucleic acid synthesis, thus affecting phage replication cycle in the host bacteria. Therefore, at the Minimum Inhibitory Concentration (MIC) dose of this antibiotic, no synergistic effect could occur. Moreover, applying combination therapy in conjunction with bacteriostatic antibiotics did not lead to a synergistic effect because these antibiotics only inhibited bacterial growth and did not reduce the number of bacteria besides avoiding the phage replication. Thus, in total, the efficiency of combination therapy of phage and antibiotics is dependent on the antibiotic resistance of examined bacteria to used antibiotic and antibiotic types (bactericide or bacteriostatic) [29, 33].

As mentioned, phage therapy can be used to inhibit MDR UPEC because, besides the appropriate antibacterial effect, it can also destroy bacterial biofilms. However, rapid emergence of phage-resistant bacteria has limited their use and it is the reason why use of phage cocktails and combination treatments should be studied further.

## *Proteus* species

*Proteus* species play a major role in UTIs and infections caused by these pathogens divided into two types: (a) hematogenous infections, e.g., systemic infections, and (b) ascending infections assisted by bacteria to be colonized step by step in the urinary tract and to reach the kidney eventually [34, 35]. Warren et al. described *P. mirabilis*, followed by *E. coli and K. pneumoniae*, as the third most common pathogen associated with the complicated UTI at a prevalence rate of 12% for infections [36]. Furthermore, CAUTI occurs in 50% of all patients catheterized for 7 days or more, and *P. mirabilis* is the predominant organism in 44% of CAUTIs [37–39]. Although treatment with antibiotics is effective in managing most of cases, rise of antibiotic resistance among CAUTI-causing bacteria, including *P. mirabilis*, makes the CAUTI treatment harder. This phenomenon highlights the importance of novel methods and phage therapy has reemerged to meet medicinal objectives in the last decade [40, 41].

**Fig. 1:**
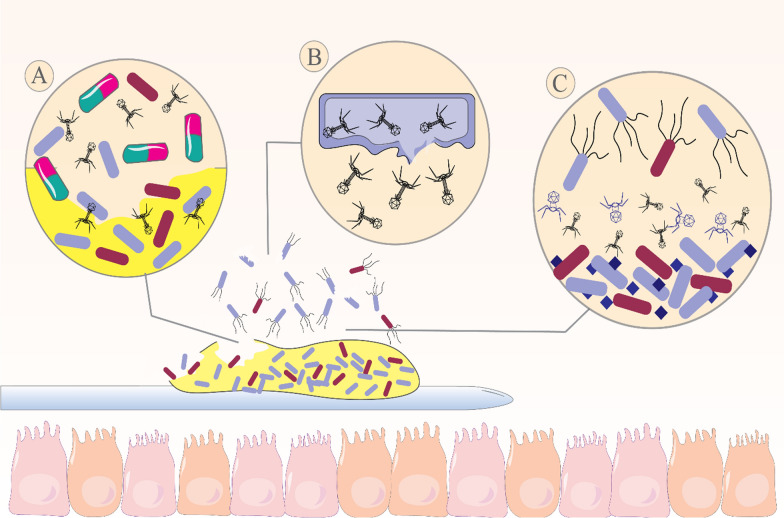
Catheter-associated urinary tract infections (UTI) and different aspects of bacteriophages therapy for inhibition and destruction of multiple-species biofilm. **a** The use of bacteriophages in combination therapies can increase the penetration of antibiotics into the deeper layers of the biofilm. **b** bacteriophages can control UTI by killing bacteria and inhibiting their virulence factors such as capsules. **c** The use of bacteriophage cocktails can be an effective to inhibit multiple-species biofilm in the UTI

In this context, two novel virulent phages including vB_PmiP_5460 and 5461 are introduced which belong to *Podoviridae* and *Myoviridae* families, respectively. These phages are used as cocktail-coated catheters to evaluate the possible effect of biofilm reduction, compared with the non-phage coated catheters. A reduction in biofilm population in phage-coated catheters is observed. Moreover, a clear tendency of phage cocktail leads to the remarkable reduction of biofilm formation in 96 and 168 h after catheterization. These effects are promising since urinary catheters remained in the patients’ bladder for a long course and the potency of the phage-coated catheters lasted for 7 days in this study [42]. Moreover, in another investigation, different degrees of sensitivity to 13 phages in 50 isolates of uropathogenic *P. mirabilis* in planktonic and biofilm forms were reported. Among the 13 phages, 39APmC32, 65APm2833 (*Myoviridae* family), and 72APm5211 (*Siphoviridae* family) were selected as cocktail components. These phages exhibited a strong anti-biofilm activity that had a better potential to inhibit biofilm formation rather than to remove mature biofilms [43]. It appears that phages can delay the formation of biofilms or even hinder their formation by inhibiting the growth of *P. mirabilis* (Fig. 1). Another good anti-biofilm feature of phages 39 and 72 A is the production of polysaccharide depolymerase, demonstrating the formation of halo around plaques. Besides, these phages eradicate more than 50% of biofilms in the investigated strains. In this way, phage-encoded polysaccharide depolymerase degrades bacterial exopolysaccharide which is the main component of biofilm matrix. It is remarkable that halos are formed by bacteria from which exopolysaccharide has been removed through excess phage enzyme released during the lysis of infected cells [44, 45]. Furthermore, applying the phage cocktails containing several phages is a reasonable solution for phages with a narrow host spectrum. In a similar fashion, in this study, phage 65 A has a low potential for destroying biofilms, but is characterized by the widest range of hosts. Authors recommend that the addition of the mentioned phage to the phage cocktail preparation could lead to increase in the number of bacteria susceptible to the cocktail, compared to preparations containing single phages. Thus, phages 72 and 39 A produce polysaccharide depolymerases that promote the penetration of phage 65 A into the deeper biofilm layer [43].

Similarly, Nzakizwanayo et al. reported three specific *P. mirabilis* phages which belonged to *Podoviridae* family. All of these phages exhibit polysaccharide depolymerase activity against their host. Furthermore, in the worst possible condition where phage is used to treat an established infection, use of a single-dose phage cocktail has remarkably (3-fold) prolonged the time taken for blocking catheters. However, the application of the same phage dose in the early phase of infection has led to complete inhibition of catheter blockage and eradication of infection. Additionally, phage treatment of biofilm formation caused a significant decrease in the level of encrustation, compared to control group. The remarkable point highlighted by this study is the importance of utilizing the phage in the early stage of infection, thus leading to full prevention of blockage. In fact, this phenomenon may be characterized by the insufficient dose of phage to deal with dense bacterial population or the development of resistance to the utilized phage [46, 47]. Furthermore, another research reported five phages with a remarkable ability to disrupt pre-formed MDR *P. mirabilis* biofilms, thus reducing the number of viable cells by 99.9% [48]. Moreover, combination therapy of ampicillin and phage vB_PmiS-TH against planktonic and biofilm forms of *P. mirabilis* recovered from UTI was used in another work. In the planktonic culture, the bacterium was highly susceptible to combination therapy. Notably, phage-antibiotic combination had the highest effect on the removal of biofilm after 24 h, which demonstrated the superiority of combination therapy to each of therapies alone in terms of efficiency. In addition, this treatment could inhibit the development of resistance mutant that develops quickly in exposure to each one of phage or antibiotics [49].

Up to 30% of all urinary tract stones (struvite) are created by *P. mirabilis* crystalline-shaped biofilms in the urinary tracts. The flow through catheters is recurrently blocked by these crystalline structures and then, bacteria embedded in crystalline biofilms become highly resistant to immune system as well as routinely used antibiotics [50, 51]. In this regard, finding a treatment to inhibit the biofilm of this bacterium can prevent CAUTI. As reported in the mentioned studies, phages can have excellent inhibitory performance against *P. mirabilis* biofilm, although further studies are required to determine how they can be used to maximize efficacy. In addition, the use of phage cocktails can target a wider range of microorganisms, and the use of a combination of phages with antibiotics increases the chances of eradicating the infection. Therefore, the use of phages to control CAUTI caused by *Proteus* species should draw much more attention.

## *Klebsiella pneumoniae*

*Klebsiella pneumoniae* are reported as one of the main opportunistic pathogens with the ability to cause hospital-acquired UTIs predominantly in the elderly, newborns, and immunocompromised individuals [52]. Dissemination of antibiotic resistance, especially to carbapenem, fluoroquinolones, and colistin, is becoming increasingly serious and only a few remaining therapeutic options remain to treat Pan-Drug-Resistant (PDR) *K. pneumoniae* infections. In this respect, the spread of PDR *K. pneumoniae* species worldwide threatens modern medicine to revert to its pre-antibiotic era [53, 54]. Thus, one of the possible options to inhibit these pathogens is to use phages due to their well-defined target spectrums and host range specificity [55]. In this section, we will discuss the specific function of phage therapy to inhibit *K. pneumoniae* in UTIs.

Sybesma et al. isolated nine *K. pneumoniae* strains (includes ESBL-producing strains) from urinary culture of different patients with UTIs and investigated the in vitro susceptibility to phages. Notably, all of these lytic phages were prepared from the phage collection of the George Eliava Institute of phage and were used in the bacterial cell lysis screening assay. The authors reported that v_BR–KpS10 could lyse all *K. pneumoniae* strains. Hence, this study proposed that existing commercial phages could be used more to control MDR *K. pneumoniae* UTI. Furthermore, the data in this study indicated that phage and antibiotic susceptibility/resistance were not related to each other and although there was resistance to various antibiotics in the *K. pneumoniae* strains, they were all lysed by one of the phages [56]. In another study, one lytic phage, KPO1K2, was isolated from effluent water to inhibit *K. pneumoniae*. This phage was a member of *Podoviridae* family with T7-like specifications and was proved stable over a wide pH range of 4–11. According to the spot test, phage was found to infect 28% (7/28) of *K. pneumoniae*. Furthermore, phage kinetics for its in vivo stability was conducted in mice. After intraperitoneal injection into mice, KPO1K2 titers in urinary bladder and kidney clearly indicated that phage accumulation occurred at higher concentrations in these two organs. Notably, in addition to *K. pneumoniae*, KPO1K2 infected *E. coli*. Since these bacteria are known for causing UTI, interestingly, both of them are sensitive to a single phage [57]. In a similar study by Zhang et al., novel phage vB_KpnP_IME279 was isolated from hospital sewage for lysis of MDR *K. pneumoniae* isolated from urine. This phage belongs to *Podoviridae* and is stable in a wide pH range between 3 and 11 and temperature range between 40 and 60 °C. It should be noted that vB_KpnP_IME279 has lytic functions against several tested *K. pneumoniae*. Furthermore, phage genome analysis reveals that the investigated phage does not contain a toxin gene, which provides safety guarantee for the future clinical treatment of this phage [58]. Therefore, the reported phages did not lose their activity even at extremes of pH; thus, it appears that they enjoy higher and longer stability in urinary bladder and kidney. In this context, these studies recommended phage therapy as a suitable candidate and a biocontrol strategy to treat MDR *K. pneumoniae*-associated UTI; however, control trails in this field are essential.

Recent researches reported *K. pneumoniae* capsule and biofilm as the most important virulence factors that have multiple functions such as adhesion to different surfaces and protection of the bacterium from lethal serum components, immune clearance, and environmental factors [53, 59]. Therefore, isolation and identification of phages that can inhibit and destroy these bacterial virulence factors is necessary to control UTI. In a recent publication, Phage 117 and Phage 31 were isolated and characterized. Then, the host range and lytic potential of these phages were tested by spotting nine carbapenamase-producing and hypermucoviscous *K. pneumoniae* (ST11 and blaKPC-2 gene positive) isolates from elderly patients with UTI. The use of each phage alone provided the potential to lyse *K. pneumoniae* strains. Subsequent culture and urine experiments with Phage 117 showed a strong lytic activity of the phages at first. Nevertheless, observation of rapid regrowth following the initial lysis suggested that phage-resistant mutants were selected among the host populations. On the other hand, the phage cocktail (117 + 31) had remarkably higher antimicrobial activity than Phage 117 alone. In this respect, the authors suggested that phage cocktail could act as a promising choice for phage therapy to control *K. pneumoniae* due to its potential ability to infect phage-resistant mutants and thus, delay the development of bacterial resistance. Although phage predation affected the assembly or synthesis of capsule expression, it had no significant effect on the biofilm formation [60]. Also, another study reported a phage, vB_KpnS_Kp13, that was effective against all Verona integron-encoded metallo-β-lactamase producing *K. pneumoniae* (ST15 and expressing the K24 capsule) originating from different hospital samples such as urine. Although this phage showed a narrow host range, no phage-resistant strains were identified in this study; thus, this phenomenon strongly suggested that the target of this phage could be related to strain survival. Of note, vB_KpnS_Kp13 had a quite efficient function in killing encapsulated bacteria; the results of analyzing its genome showed that the specific feature of the capsule depolymerase, encoded by ORF2, of this phage provided the mentioned function. Furthermore, the phage significantly degraded biofilm and reduced the biomass by ~ 73% in 48 h post-treatment [55].

In this respect, in another study, a phage 0507-KN2-1 was isolated for a new capsular type of *K. pneumoniae* (KN2) UTI isolates. This phage was categorized as a member of the *Myoviridae* phage family and the analysis of the genome indicated a putative polysaccharide depolymerase encoded by 3738-bp gene. A recombinant form of this protein was produced and was analyzed for confirmation of its enzymatic activity and specificity to capsular polysaccharides. Then, in spot tests, this purified depolymerase led to decapsulation of *K. pneumoniae* strains. Notably, this protein was specific to KN2 capsular polysaccharides and was not able to induce digestion-like spots on *K. pneumoniae* strains with another type of capsule or KN2 capsular polysaccharide mutant [61]. Furthermore, in another work, *Siphoviridae* phage TSK1 showed a potent lytic activity against *K. pneumoniae*. This phage had a narrow host range and enjoyed the highest stability at pH 7 at a temperature of 37 °C. Besides, post-treatment with TSK1 caused a reduction in *K. pneumoniae* biofilms biomass (of different ages). Pre-treatment of *K. pneumoniae* biofilm with TSK1 decreased biomass > 99 % in the first 24 h of incubation. Therefore, this study recommended that due to TSK1 stability at alkaline pH, this phage can be employed as a medicinal agent for *K. pneumoniae*-mediated UTIs, while it is not suitable for direct oral administration. Also, due to the higher ability of TSK1 to destroy bacterial biofilm, this phage can be utilized in the impregnation of urinary catheter to hinder CAUTIs [53].

It seems that phages and recombinant depolymerase can be used as therapeutic strategies to control *K. pneumoniae* UTI, given that phages can prevent or destroy the most important virulence factors of this bacteria such as capsules and biofilms through capsule-polysaccharide specific depolymerase. Of note, this enzyme is required for degradation of capsule and adsorption onto the host cell. Consequently, following the degradation of capsule, the exposure of outer membrane components to phage for better accessibility occurred. On the other hand, according to findings, phage depolymerase acts specifically against a specific capsule type and isolated phages usually have a narrow host range and can only be used for a specific bacterial strain. Therefore, phage can be employed in combination with other phages in phage cocktails and with antibiotics to combat *K. pneumoniae* in UTIs.

In general, in addition to UPEC, *Proteus* species, and *K. pneumoniae* that have been studied further in the field of phage therapy to control UTIs, other researchers have investigated the effect of phages on uropathogenic bacteria. In this manner, Table 1 lists the recent related studies that used phages to inhibit different uropathogenic bacteria.

## Multi‐species cocktails

The prevalence of antimicrobial resistance causes obstacles to a successful treatment and also, biofilm-based infections create even more challenges these days; therefore, phage therapy could be a useful choice in combating MDR uropathogenic bacteria such as *Proteus* spp. and *E. coli*. In this manner, a study investigates the activities of three commercial phage cocktails (Septaphage, PYO and INTESTI) against 70 MDR *E. coli* and 31 *Proteus* spp. (including 15 MDR strains) collected from human and non-human origins. These cocktails were produced by Georgian institutions including PYO and INTESTI from Eliava BioPreparations and Septaphage from Biochimpharm. The percentages of the susceptibility of *E. coli* strains to PYO, INTESTI, and Septaphage were 61.4%, 67.1%, and 8.6%, respectively. Also, the susceptibility of *Proteus* spp. to PYO, INTESTI, and Septaphage was 29.0%, 38.7%, and 19.3%, respectively [62]. Interestingly, Septaphage almost represented no activity against the investigated strains. This difference among biopreparations results from different production methods and contents that caused insufficient phage titer of the final product [63]. Therefore, it appears that the function of commercial phage cocktails to inhibit MDR bacteria is more limited. This narrow activity could result from the absence of specific phages targeting contemporary MDR strains which are spreading in different settings. Therefore, the specific phages for the emerging MDR bacteria should be isolated, well characterized, and then, integrated into conventional biopreparations [62, 64]. Because it is only in this case that commercial phage cocktail can be used to treat MDR UTI around the world.

**Table 1:** Recent studies used phage therapy to inhibit the most common uropathogenic bacteria

Uropathogenic bacteria	Properties	Phage	Outcome	Reference
*Enterobacter cloacae*	MDR	E-2, E-3 and E-4 were isolated from waste water	The growth of the bacteria was inhibited by the three phages. Notably, the use of cocktails with two or three phages was significantly more effective than each one alone. In urine, the inactivation was still effective	[65]
*Enterococcus faecalis*	Clinical isolates	vB_EfaS_GEC-EfS_3 (*Siphoviridae*) isolated from sewage	While phage was able to infect a broad range of strains of the same species as the host species from which they were isolated, they were unable to infect other host species tested	[66]
*Enterococcus faecalis*	VRE	vB_EfaS_HEf13 (genus *Sap6virus* in the family *Siphoviridae*) was collected from a local sewerage system	The lytic activity of phage HEf13 at various multiplicities of infection consistently inhibited the growth of diverse clinical isolates of *E. faecalis* without any lysogenic process	[67]
*Streptococcus mitis*	Clinical isolates	vB_SmM_GEC-SmitisM_2 (*Myoviridae*) isolated from sewage	This phage was able to productively infect 9 of 16 *S. mitis* strains, but none of the other species in our collection	[66]
*Staphylococcus saprophyticus*	MDR Clinical isolates	vB_SsapS-104 (*Siphoviridae*) was isolated from hospital wastewater	This phage represented high anti-bacterial activities against *S. saprophyticus* isolates in vitro, as it was able to lyse 8 of the 9 clinical isolates (88.8%). Notably, no lytic activity was observed on some other pathogenic bacteria tested	[68]

MDR: Multi-drug resistant; VRE: vancomycin-resistant enterococci

## Phages inhibition for other uropathogenic bacterial biofilm

As discussed in the previous sections, phages have the ability to destroy the biofilm of bacteria that cause UTI; however, during the course of this infection, different bacteria forms mixed-species biofilm on in-dwelling medical devices or host organs. So, using a specific phage of bacterial species cannot completely eradicate the infection [69].

In a recently published paper, a cocktail consisting of MDR *A. baumannii* infecting phages (Aba 1–6) with highly lytic activities varying from 56 to 84% was used to destroy the bacterial biofilm in combination with antibiotics in human urine. The usage of all phages significantly reduced *A. baumannii* biofilm biomass, and Aba-1 appeared to be having the highest antibiofilm activity in the urine condition. Furthermore, the use of phage cocktail in combination with trimethoprim/sulfamethoxazole (SXT) and ciprofloxacin led to biofilm biomass reduction rates of 94.3 and 93.3%, respectively. Furthermore, in most cases synergistic effect also causes the limitation of persister cell regrowth. Of note, combination therapy with gentamicin, tobramycin, imipenem, and meropenem similarly induced a more significant reduction in *A. baumannii* biofilm biomass than the phage cocktail-treated control. On the other hand, combination therapy with colistin did not present the same expected outcome as its mentioned counterparts mentioned above. The synergistic impact can be clarified by the antibiotics mode of activity. Antibiotic-related changes within the bacterial cell morphology empowered fast phage development and cell lysis. Besides, the set of phage lytic enzymes, particularly those responsible for local peptidoglycan degradation, could enhance antibiotic penetration through biofilm matrix. On the other hand, combination of phage and colistin did not have synergistic effect because colistin destabilized the bacterial cell membrane and could limit phage propagation. In addition, overexpression of the efflux pump and autoinducer synthase AbaI that accelerate the synthesis and transport of acylated homoserine lactones can be related to the increase of biofilm formation. Therefore, combined treatment can destroy the biofilm of MDR *A. baumannii* in UTI through such mechanisms as enzyme-mediated permeabilization of membranes, digestion of the biofilm matrix, and enhancement of antibiotics activity. In addition, phage therapy can target persister cells through antibiotic treatment. Nevertheless, this activity may depend on the type of bacterial pathogen and the antibiotic used [10, 70].

In another investigation by Yang et al., PHB08, a virulent *E. faecalis* phage, and its endolysin (lys08) demonstrated antibiofilm activity against *E. faecalis* biofilms isolated from UTI. Different analyses revealed that the mentioned phage belonged to *Siphoviridae* family and exhibited great stability at different temperatures (4–60 °C) and pHs (between 5.0 and 9.0). Furthermore, this phage infected 79% (15/19) of the *E. faecalis* clinical isolates tested, but had no effect on other bacterial species such as *E. faecium*. Both phage and its endolysin remarkably decreased bacterial biofilm density. So, this study also suggested phage and endolysin as possible biocontrol agents that could destroy the biofilm formation of *E. faecalis*. Additionally, high stability of phages in different environmental conditions can increase their use in UTI [71]. Another interesting study on two-species biofilms investigated the effectiveness of hydrogel silicone urinary catheter treated with phage cocktail in inhibition of biofilm formation by a mixture of *P. mirabilis* and *P. aeruginosa* in an in vitro model using artificial urine. The observations indicated that pre-treatment with phage led to reduction of *P. mirabilis* biofilm number by > 2 log_10_ CFU/cm^2^, while this value was 4 log_10_ CFU/cm^2^ for *P. aeruginosa* over 48 h. Notably, the presence of *P. mirabilis* always causes an increase in lumen pH from 7.5 to 9.5 and such a high pH has no inhibitory effect on the function of phages in the catheter model. On the other hand, survival of bacterial population was reported for both pathogens in the anti-biofilm assay. However, plaque formation observed on the plates with the recovered adherent bacteria implied that large fractions of these bacteria were not truly phage resistant [72]. This phenomenon may be related to metabolic alternation of bacteria in biofilm community, especially cells in the dipper layer of biofilm that did not support phage replication following infection. Another alternative explanation could be “spatial refuges” in which resistant bacteria create a physical barrier for susceptible cells against phage invasion [44, 72]. Therefore, the authors proposed that it was possible to use phages in indwelling urinary catheters with high pH conditions. Additionally, phages can be used to inhibit multiple species in mixed-species biofilms without interfering in the lytic capacities of the other phages. These findings demonstrate the possibility of applying a phage cocktail to CAUTIs because the introduction of a foreign body in the urinary tract facilitates higher microbial colonization and mixed-species biofilm formation.

In another research, it was also reported that pre-treating urinary catheter with *P. aeruginosa* specific phage ΦE2005-A and *E. coli* HU2117 had a synergistic protection against the formation of *P. aeruginosa* biofilm on catheters. In this regard, silicone catheter segments were exposed to phage plus *E. coli* and challenged by *P. aeruginosa*; then, incubation occurred in the human urine sample up to 72 h. The result showed that this pre-treatment led to the lower adherence of 4 log_10_ unit of *P. aeruginosa* to the catheter than no pre-treatment. It is worth mentioning that due to the specificity of the phage host, the presence of special *Pseudomonas* phage did not considerably interfere in *E. coli* HU2117 communities [21]. These discoveries revealed that the combination of phage and probiotic bacterium interference would lead to the attenuation of biofilm formation by *P. aeruginosa* on urinary catheters. This phenomenon results from the fact that the use of specific *P. aeruginosa* phage reduces the initial population of this bacterium, a process that allows the *E. coli* biofilm to be established easier. Therefore, mixed biofilm does not form because the combined effect of microbial competition and lytic phage infection may lead to the predominance of one species in the biofilm. However, the use of phages and *E. coli* alone did not show good outcomes Because it seems that *E. coli* was unable to outperform *P. aeruginosa* and using the phage solitarily was obviously insufficient to destroy all *P. aeruginosa* cells prior to mixed biofilm formation on the catheter [21, 73].

The definite role of catheter-associated biofilms in UTI is poorly known; however, there is evidence that this kind of biofilms has a major role as the stable reservoir of uropathogenic microorganisms, which are resistant to antimicrobial and it is difficult to eliminate them even with the catheter removal. Therefore, prevention and removal of bacterial biofilm is very important in the treatment of UTIs. In this context, phages have been reported as one of the most significant candidates for inhibiting bacterial biofilm. However, bacterial biofilms are usually produced by several different pathogens and cannot be completely eradicated using a specific phage (Fig. 1) [74]. Therefore, as mentioned in the above studies, the combined use of several different phages as phage cocktail, the combined treatment of phages and antibiotics, and the use of probiotics along with phages can help inhibit the mixed biofilm further. Furthermore, it may be useful to investigate the possible synergistic interactions between phage cocktails and other antimicrobial strategies such as biofilm inhibitors, catheters with more routine antibacterial substances or bacterial interference. Notably, despite the use of different combination therapies, phage-resistant bacteria are still reported in studies; however, with the use of combination therapies, their population is greatly reduced. Therefore, it is very important to identify more effective strategies to kill all bacteria in the biofilm and prevent the development of resistance to phages. In doing so, the true value of these treatments is determined when used in in vivo and clinical studies.

## In vivo and clinical use of bacteriophage for the treatment of UTIs

As fully discussed in the previous sections, the use of phages to inhibit bacteria that cause UTIs has been reported in many in vitro studies. On the other hand, phage therapy has not been well received in clinical and in vivo researches. Nonetheless, we will discuss clinical and in vivo studies that have used phages to control and treat UTIs.

In the case of a 60-year-old male patient who underwent kidney transplantation, multiple chronic UTIs with an MDR *K. pneumoniae* were reported. During each hospital stay, the patient was treated for 12–14 days with meropenem alone or meropenem with colistin and it yielded rapid, yet temporary, resolution of infection symptoms. After 10 severe cases of UTI with hospitalizations, phage therapy was used to treat the infection. In this regard, the intra-rectal application (10 ml twice daily) of the specific phage preparation against the MDR *K. pneumoniae* strain was used; however, after five days, the patient’s condition worsened and ultrasound examination showed polycystic kidneys. Therefore, a combination of meropenem and phage was used and after 18 days of this treatment, progressive remission of clinical signs was observed. Due to multiple recurrent infections and unsuccessful outcomes, the patient was selected eventually for a planned resection of his left polycystic kidney [75]. In this patient, the main source of infection was cysts in the left kidney that caused several therapeutic failures; surgical intervention achieved the best therapeutic outcomes. Furthermore, due to the combined use of phage and antibiotics for the treatment of the patient, it is difficult to evaluate the exact function of phage therapy, but it seems that the infection in the transplanted kidney could be controlled through phages. On the other hand, shortly after the use of phage therapy, it is possible that phage may have facilitated the selection of phage-resistant bacteria. Thus, the use of phage cocktails or combination therapies with antibiotics can be helpful [76]. Notably, *K. pneumoniae* isolated from the patient were completely sensitive to the colistin; however, after 12 days, antibiotic therapy stopped on the suspicion of nephrotoxicity. However, long-term (29 days) use of phages and phage lysate application were safe, particularly in relevance to the long-term kidney function and allograft tolerance.

Phage therapy was also used in another 63-year-old female patient with recurrent UTI caused by Extensively Drug-Resistant (XDR) *K. pneumoniae*. It is noteworthy that, this bacterium was completely resistant to all tasted antibiotics except polymixin B and tigecycline. This patient was treated by antibiotics, but due to antibiotics utilization, complication became increasingly severe. Hence, she agreed to a phage therapy clinical trial and then, phage cocktail consisted of five lytic phages, used for the first round by bladder irrigation. This phage therapy regimen (Cocktail I) failed to completely kill the bacteria, and phage-resistant bacteria were isolated. In this respect, another cocktail (Cocktail II) was used, which included specific phages against bacteria isolated from the patient. Unfortunately, phage-resistant bacteria were isolated again. Thus, it was hypothesized that the combined use of phages and non-active antibiotics against bacteria could be helpful and in this way, the combined use of bladder-irrigated cocktails (Cocktail III) and oral SXT showed strong synergistic effect and the recurrent UTI symptoms subsequently disappeared [20]. Therefore, the findings of this study suggested combining the phage cocktail with antibiotic in the treatment of recurrent UTI in order to lower the risk of regrowth of phage-resistant mutants. It seems that the rapid onset of phage-resistant strains has been due to the poor functioning of the patient’s immune system, given that a proper immune system function is essential to the clearance of bacterial infection [77]. However, more molecular studies are required to understand the exact mechanism of resistance to phage cocktails.

A recently published study by Ujmajuridze et al. evaluated different characteristics of phage therapy such as feasibility, tolerability, safety, and clinical/microbiological outcomes in patients with UTIs. In this way, PYO phage (phage cocktail consisting of phage lines is activated against a broad spectrum of uropathogenic bacteria) produced by Eliava was used. After in vitro phage sensitivity test, the hospital was informed of PYO phage solution to initiate the clinical analysis. After Transurethral Resection of Prostate (TURP), suprapubic and transurethral catheters were inserted to maintain phage cocktail irrigation. Then, after one to two days, the transurethral catheter was removed, while the suprapubic catheter was kept in place for another 7 days to facilitate the adapted phage cocktail instillation. Total sensitivity of 41% was reported in in vitro analysis. Besides, in in vivo pilot series, 67% (6/9) of patients’ bacterial titers were reduced after phage treatment. Notably, no phage-associated side effect was reported. So, the authors suggested that phage therapy could be safe and effective in UTI treatments [78].

In a study conducted by Nishikawa et al., UPECs were injected transurethrally into the mouse bladder. Then, purified phages T4 and KEP10 (*Myoviridae* family) were immediately injected into the peritoneal cavity of mice to treat UTI. The findings manifested that phages could save a large percentage of mice (up to 90%) from death through a dose-dependent infection. On the other hand, all mice in the control group that were not treated after infection died within 3 days. Notably, no specific side effects were reported for any of the phages. Moreover, the results illustrated that these phages moved rapidly into the bloodstream and disseminated into internal organs and they remained stable for 24 h at most in the human and mouse urine samples at 37 °C. Therefore, the authors reported T4 and KEP10 as suitable therapeutic phages for treating lethal UTI [79]. Finally, in another in vivo study on mice, UTI was induced by transurethral application of *Cronobacter turicensis*. At the same time, specific phages against this bacterium were administered intraperitoneally. The authors reported that phage therapy decreased the number of bacteria in the kidney by 70%. Phage therapy reduced the higher levels of malondialdehyde by 39% without any effect on the antioxidant status. Notably, there was no significant difference in *Cronobacter* colonization of the bladder between infected and phage treated groups. Furthermore, the expression of pro-inflammatory cytokines such as monocyte chemoattractant protein-1 and tumor necrosis factor-alpha increased due to the infection, while it was attenuated by phage therapy. The authors report that differences in the function of phages in the kidney and bladder can be related to the kinetics of phages after intraperitoneal administration, which could be improved by choosing another application route [80].

Therefore, the finding of various studies represented that phage therapy could be used to control UTI caused by MDR bacteria, although there are still many limitations in this treatment regimen. The administration of phages varied in different studies and there is still no comprehensive guideline on how to use them to ensure the highest clinical performance. Therefore, more clinical trials are needed to fully evaluate the safety and efficiency of potential therapeutic applications in vivo. Further, when a specific phage is used alone, the risk of development of phage-resistant mutant is very high. In this regard, the use of phage cocktails can be an effective solution because different phages use different receptors to minimize the development of resistance and widen the host range, which is important in the case of narrow host range infecting phages. However, as reported in the mentioned studies, resistance to cocktails has also occurred, but their molecular mechanisms have not yet been elucidated. Thus, the combined use of phage cocktails and different antibiotics increases the chances for controlling the UTI. At last, those antibiotics that are effective against uropathogenic bacteria such as polymixin and tigecycline are not usually used for a long time due to their many side effects, especially nephrotoxicity, although various studies have not reported the specific side effects of phages, which could lead to their widespread use in treatment of UTIs.

## Conclusion

MDR Uropathogenic bacteria have reduced the utility of chemical antibiotics in clinical settings. Moreover, these antibiotics are cytotoxic to not only pathogens, but also health-beneficial commensals. In this regard, phages and their derivatives were reported as an alternative strategy for the treatment of drug-resistant UTI. Recent studies found that phages, in addition to inhibiting uropathogenic bacteria, could destroy their biofilm. Besides, the safety profile of these microorganism seems to be far better than antibiotics. Therefore, researchers suggested phage therapy as a mean for prevention and treatment of UTI and further spread of MDR uropathogenic bacteria. However, phage-resistant strains still occur despite the use of phage cocktails and combination therapy. In this context, more fundamental studies are required to determine the phage–host interactions and the phage potential to control UTI. Additionally, most of phage therapy data were obtained from in vitro studies and the major limitation was lack of appropriate clinical research. So, further clinical trials (double-blind and placebo-control) are needed to investigate dose, best routes of administration, frequency and duration of phage therapy for inhibition and treatment of UTI.

## Data Availability

The authors confirm that the data supporting the findings of this study is available within the article.
